# Case studies on the formation of chalcogenide self-assembled monolayers on surfaces and dissociative processes

**DOI:** 10.3762/bjnano.7.24

**Published:** 2016-02-17

**Authors:** Yongfeng Tong, Tingming Jiang, Azzedine Bendounan, Makri Nimbegondi Kotresh Harish, Angelo Giglia, Stefan Kubsky, Fausto Sirotti, Luca Pasquali, Srinivasan Sampath, Vladimir A Esaulov

**Affiliations:** 1 Institut des Sciences Moléculaires d’Orsay, UMR 8214 CNRS-Université Paris Sud, Université Paris-Saclay, F-91405 Orsay, France; 2 Synchrotron Soleil, L’Orme des Merisiers, Saint-Aubin, BP 48, F-91192 Gif-sur-Yvette Cedex, France; 3 Dipartimento di Ingegneria ‘E. Ferrari’, Università di Modena e Reggio Emilia, Via Vignolese 905, 41125 Modena, Italy; 4 Acharya Institute of Technology, Bangalore 560 107, India; 5 CNR-IOM, s.s.14, km 163.5 in Area Science Park, 34012 Trieste, Italy; 6 Physics Department, University of Johannesburg, P.O. Box 524, Auckland Park 2006, South Africa,; 7 Department of Inorganic and Physical Chemistry, Indian Institute of Science, CV Raman Avenue, Bangalore 560 012, India

**Keywords:** copper, nickel, palladium, reactivity, selenol, selenophene, self-assembly, thiol, thiophene

## Abstract

This report examines the assembly of chalcogenide organic molecules on various surfaces, focusing on cases when chemisorption is accompanied by carbon–chalcogen atom-bond scission. In the case of alkane and benzyl chalcogenides, this induces formation of a chalcogenized interface layer. This process can occur during the initial stages of adsorption and then, after passivation of the surface, molecular adsorption can proceed. The characteristics of the chalcogenized interface layer can be significantly different from the metal layer and can affect various properties such as electron conduction. For chalcogenophenes, the carbon–chalcogen atom-bond breaking can lead to opening of the ring and adsorption of an alkene chalcogenide. Such a disruption of the π-electron system affects charge transport along the chains. Awareness about these effects is of importance from the point of view of molecular electronics. We discuss some recent studies based on X-ray photoelectron spectroscopy that shed light on these aspects for a series of such organic molecules.

## Introduction

In recent years research related to various applications such as catalysis, sensor development, hydrogen storage, thin films, and molecular electronics has focused on the study of self-assembled monolayers (SAMs) with different combinations of molecular architecture, and in particular, different molecule anchoring head groups. The latter determines the binding to the substrate and plays an important role in defining the molecular ordering and electronic coupling, which determines the charge flow between the molecular components and the substrate electrode. Much work on various aspects of assembly and its uses has been performed with sulfur head group (thiol) molecules [1–27], but interest in other head group atoms such as C [28–29], N [30] and other chalcogenides has also received increased attention. In particular, selenium head group SAMs have attracted much attention and significant research has resulted [31–43] for substrates such as Au and Ag. There is considerable discussion in the literature about the strength of the head group substrate bond [14,34,41] and whether or not it provides a better conductance pathway than sulfur. Besides the case of self-assembly on bulk metal surfaces, the knowledge of the physics and chemistry of chalcogenide SAMs on metal nanoparticle surfaces is also very important as they are widely used in different areas.

In this paper, we focus on recent work where the interaction with the substrate is strong and can lead to dissociative processes. This is, for instance, the case of copper and transition metals (Ni and Pd), which are characterized by a greater reactivity than gold. Thus, in the case of thiophene, dehydrogenation and desulfurization is well known to occur on transition metal surfaces [44–45]. A few years ago, the research groups of Nuzzo [46], Whitesides [46] and others [48] noted that for alkane thiol SAMs, the initial desulfurization occurs via S–C bond scission. This leads to the formation of a sulfidic interface layer, upon which a more or less ordered molecular layer can eventually form. This was noted for the case of alkanethiol SAMs on Pd and it was shown that this led to interesting consequences, such as greater resistance to corrosion by chemical etchants [46–47]. In biotechnology applications, a greater resistance to invasion by cells was observed in this case as opposed to the case of the same SAMs on gold [46–47]. Similarly, in some examples of thiol adsorption on Cu, there exists evidence [26] of S–C bond scission with sulfur remaining on the surface.

It is clear that such processes strongly affect the interface properties and in particular the characteristics of charge transport through such a sulfidic interface layer would be strongly affected.

In the context of the use of nanoparticles in various applications [49–52] such as in catalysis, sensing or hydrogen storage, capping the nanoparticles using thiols leads to important questions related to the nature of the interface layer. For instance, in the case of palladium nanoparticles there has been a controversy [53–55] about whether the thiol was adsorbed on the metal core or rather if the metal core was capped by a PdS layer on which the alkanethiol was formed. This has obvious implications in hydrogen storage applications, for example, where hydrogen permeation into Pd [52] is inhibited by a sulfidic layer.

While it might not seem surprising that such dissociative processes may take place on reactive substrates, it is noteworthy that this has been invoked for the case of thiol and selenol SAMs on Au [30], as well as in the case of ultrathin layers of thiophene derivatives on Au. In the latter case, S–C bond scission occurs, leading to the opening of the ring and observation of thiolate species [52–63]. The loss of aromaticity and planarity can thus occur and this interrupts the π-electron system and impairs charge transport along the chains. There are indications of the appearance of atomic S on the surface [60–61,63], i.e., complete desulfurization for these thiophenes.

Finally, experiments show that the presence of these reactive channels depends on the preparation method, e.g., vacuum versus liquid phase adsorption [56–58], or also deposition onto bulk metal versus evaporation of electrodes onto a molecular layer [64–65]. This point is of much importance when creating contacts to these organic species.

It should be noted that in many cases the conclusions of the above mentioned investigations of dissociation processes in thiol self-assembly rely on the knowledge of the characteristic S 2p core level binding energies (CLBEs) for atomic S adsorption and the thiolate sulfur. These are usually deduced from X-ray photoelectron spectroscopy (XPS) studies. Thus, it was noted in the Pd case that the S 2p region spectra differed significantly from those observed for Au and Ag substrates. This is because of contributions from both atomic and thiolate sulfur. The situation is in general complicated by the fact that molecular adsorption can occur on alternative or “unusual” adsorption sites, in the sense that they are not observed in well-ordered SAMs [27]. For these cases, it has been observed that the thiolate CLBE can be close to that of atomic S on Au and Ag. This engenders serious ambiguities. Interestingly, on transition metals such as Rh, W and Ni, there are observations of multicomponent S 2p spectra [66–68], which could be due to some type of interface sulfide layers. Understanding the interfaces in these situations is thus very important. A good knowledge of monolayer atomic chalcogen adsorption is obviously an important prerequisite, as well as information regarding the CLBEs of S 2p (Se 3d, etc.) for such coverages is essential. This is still frequently unavailable, and obtaining precise spectroscopic information on this aspect is an essential complement to the investigation of other characteristics of molecular adsorption. Furthermore, there is currently much interest in nanostructured metal chalcogenides and ultrathin films, and in this context, this data is also of significant relevance.

**Figure 1:**
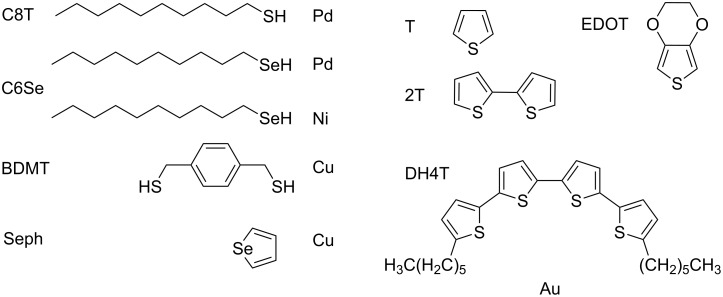
The main molecule–metal combinations discussed in this report: alkane chalcogenides (CnT), 1,4-benzenedimethanethiol (BDMT), selenophene (Seph), 3,4-ethylenedioxythiophene (EDOT), thiophene (T), bithiophene (2T), and α,ω-dihexylquaterthiophene (DH4T). Other examples of EDOT family compounds are mentioned later.

In this progress report, we discuss recent work performed mainly in our groups on chalcogen SAM formation on reactive substrates, for which there exists few studies. We summarize selected, recently published work, as well as new data for alkane and aromatic chalcogenide molecules on Au, Cu, Pd and Ni surfaces. The main systems discussed are schematized in Figure 1. We chose alkane chalcogenide molecules that have been extensively studied on Au and a few aromatic molecules such as benzenedithiol, thiophene derivatives and selenophene that are of interest in molecular electronics.

## Experimental

The experimental procedure has been outlined in detail in previous publications [22–24,26–27,43,48,69] and here we provide only essential points about new experiments relevant to this report.

### Sample preparation

Sulfur and selenium adsorption was performed, as in an earlier study [69], by immersion into a 0.1 mM Na_2_S or Na_2_Se solution in 0.1 M aqueous NaOH. Na_2_Se, alkanethiols, thiophenes and selenophene were purchased from Sigma-Aldrich and α,ω-dihexylquaterthiophene (DH4T) from SYNCOM, and all were used as supplied. DC6DSe ((CH_3_CH_2_)_5_Se)_2_ was synthesized according to the procedure described in an earlier publication [43] and in Supporting Information File 1.

Adsorption of DC6DSe was performed with a 1 mM solution in ethanol. It is known to lead to hexaneselenol adsorption on Au [37]. Thiophene and bithiophene adsorption was performed from a 1 mM ethanolic solution, whereas for DH4T, adsorption was performed from a 1 mM solution in dicholoromethane. In all cases a 24 h immersion time was used. Selenophene adsorption was performed from pure selenophene for 1 h.

The Au samples were prepared by evaporation onto hot mica that had been degassed for three hours at 300 °C. Au deposition was done at this temperature and then a brief heating to 550 °C was performed. The Cu(111), Pd(111) and Ni(111) monocrystals were purchased, oriented and polished, from Mateck or from the Surface Preparation Laboratories. In situ surface preparation was performed as usual by cycles of sputtering and annealing, and the surface cleanliness and crystallinity was checked by XPS and low energy electron diffraction (LEED).

The prepared samples were extracted from the ultra-high vacuum preparation chamber under N_2_ flow and immediately immersed into the solutions. Thereafter, they were rinsed in the corresponding solvents and dried by N_2_ gas. The samples were then immediately transferred into the analysis chamber.

### Photoemission

The photoemission experiments were performed mainly on the BEAR beamline [22–24,26–27] at the Elettra (Trieste, Italy) synchrotron and the TEMPO beamline [43,48,69] at the Soleil (St. Auban, France) synchrotron. The experimental procedures are described in the indicated references. The energy resolution was about 50 meV and 200 meV on the TEMPO and BEAR beamlines, respectively. The measurements on thiophenes were performed using a VG-Microtech K-Alpha spectrometer incorporating a monochromatic X-ray source with an Al anode at the Orsay campus. In this case, the energy resolution was 500 meV.

The binding energy in the XPS spectra was calibrated with respect to the Au 4f_7/2_ peak, set at 84 eV. The calibration error is estimated to be 50 meV at TEMPO and about 100 meV at BEAR. With some exceptions (indicated later in the text), we used a photon energy corresponding to a final kinetic energy of ≈100 eV in order to maximize surface contributions.

Because X-ray irradiation is known to lead to alterations in the organic layers, particular care was taken to distinguish this effect. This was done by comparing the spectral shapes for successive scans and performing measurements on several points on the sample. A detailed discussion of this can be found in the original publications [26,48] and in the selenophene section below.

NEXAFS spectra presented here were recorded in partial yield mode by measuring the carbon Auger signal that appears consistent with the total yield measurements. We have used synchrotron light with 100% horizontal linear polarization. To probe the molecule orientation over the surface, we varied the polar angle by rotating the sample around the *z*-axis (the polarization is parallel to the surface plane for Θ = 90°).

## Results and Discussion

### 1,4-Benzenedimethanethiol adsorption on copper surfaces

Dithiol SAMs have attracted attention in particular because the two thiol ends can be used as linkers between metal electrodes and thus metal–organic heterostructures can be constructed [70–74]. 1,4-Benzenedimethanethiol (BDMT) has been the object of several investigations on gold [21–25,75] and this dithiol was used in one of the first studies of molecular conductance [70]. Many of these studies use gold electrodes. It was interesting to extend these investigations to another prototype electrode metal: copper. This prompted the work described below. Studies of alkane and phenyl thiols do exist and they conclude that an ordered thiol layer is formed [76–78].

BDMT evaporative adsorption was studied on Au(111), Cu(100) and Cu(111) surfaces by Alarcón et al. [25,79] using time-of-flight ion scattering [80], which allows the study of the surface composition without inducing noticeable damage. It was observed that in the case of the copper surfaces, at the onset of adsorption, a substantial amount of sulfur on the surface appeared, while the carbon concentration remained small and increased only after addition of a much larger amount of BDMT. This could be interpreted as initial BDMT decomposition due to S−C bond scission that led to the presence of atomic S on the surface. This was surprising since such S–C bond scission was not observed for room temperature adsorption of alkanethiol and phenyl thiol [76–78].

**Figure 2:**
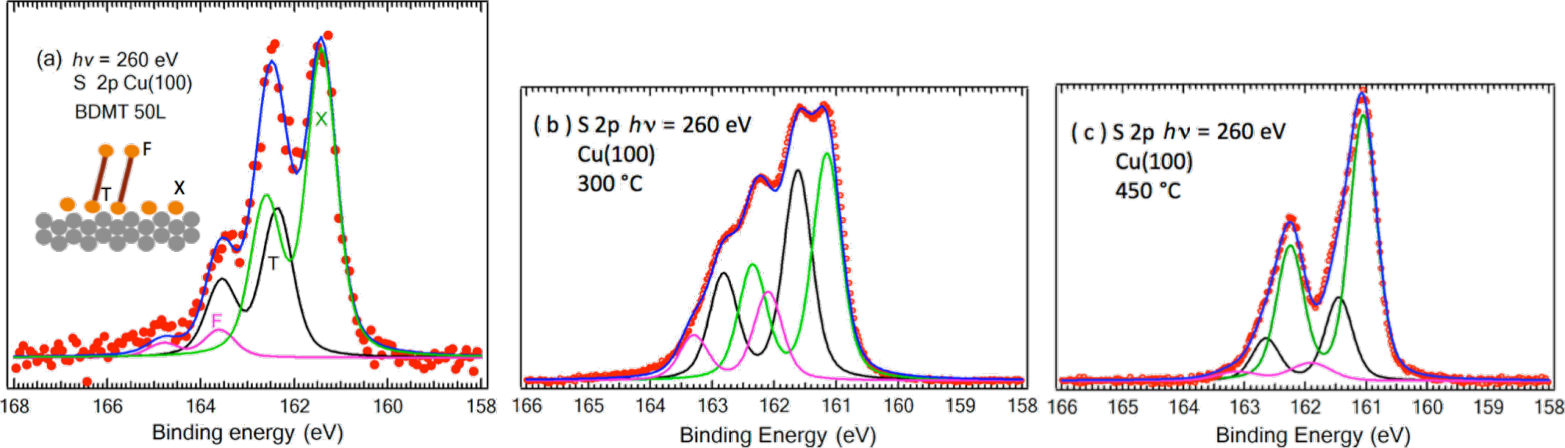
(a) S 2p XPS spectra for a small dose of BDMT evaporated [26] onto Cu(100). (b,c) S 2p spectra for S adsorption onto Cu(100) [82] from Na_2_S. The solid lines are fits using Voigt contours and the appropriate spin–orbit splitting of the S 2p_3/2_ and S 2p_1/2_ components. Figure adapted with permission from [82], copyright 2014 American Chemical Society.

To verify this, a high-resolution XPS study was performed [26] and S 2p spectra were recorded from low sub-monolayer coverage to very high exposures. Figure 2a shows the S 2p spectrum after exposure of a Cu(100) surface to 50 langmuir of BDMT. For dithiol adsorption on Au, in the case of well-ordered SAMs, one observes two doublets with the S 2p components located at about 162 eV and 163.1 eV. The former corresponds to thiolate sulfur (bound to Au) and the latter to the sulfur of the “free” SH end group on the top of an upright SAM. In the case of Cu at the lowest exposure, a strong component at 161.4 eV is observed. This could be due to an alternative adsorption site or to atomic S from dissociation. To clarify this, one needs information on the CLBEs for atomic S adsorption on Cu. Atomic S adsorption on Cu(100) and Cu(111) surfaces [81–87] leads to rather complex surface structures explored in several STM studies [81,84–85] and these are still being actively studied [82,86–87].

Although the S 2p binding energies for bulk copper sulfide are known, with a rare exception [83], there was previously not much information on CLBEs for sub-monolayer chemisorbed phases. A detailed photoemission study was therefore performed [82], revealing multicomponent S 2p spectra with different CLBEs corresponding to differently coordinated S atoms for different coverages (Figure 2b,c). The spectra in Figure 2b,c were taken after a pristine UHV prepared Cu surface was dipped into a Na_2_S solution and then annealed to the indicated temperatures. This leads to the appearance of well-ordered structures that are identifiable by LEED. The 161.4 eV CLBE corresponds closely to one of the observed components for BDMT adsorption. It was also found [26] that annealing the BDMT-exposed Cu results in the molecular decomposition and appearance of residual S on the surface with this binding energy.

A careful analysis of the relative intensities of the C 1s and S 2p peaks in BDMT adsorption shows [26] that at this low 50 langmuir exposure, the amount of carbon present on the surface is much lower than could be expected. These measurements thus indicate that in the initial stages of adsorption, S–C bond scission occurs, leading to sulfidation of the Cu surface. Thereafter, when the surface is passivated, molecular adsorption occurs. The remaining molecular fragment after dissociation appears to leave the surface. Possibly [26], the loss of this fragment could be mediated by a H-atom transfer, leading to the formation of a less reactive, CH_3_-terminated species and the sulfurized (Cu–S) surface:

Cu + HS–CH_2_–R–CH_2_–CH_3_ → Cu–S + CH_3_–R–CH_2_–CH_3_

### Alkanechalcogenides on Pd

The formation of alkanethiol SAMs on Pd was reported by several authors [46–47]. The research groups of Nuzzo and Whitesides concluded that alkanethiol SAMs [46–47] were not formed directly on the Pd(111) surface, but rather on a PdS interface layer. Similar conclusions were recently reached by others [88].

Let us first look at the adsorption of sulfur on Pd. Previous studies [81,89–92] showed the existence of several structures in the sub-monolayer range, namely: the (√3 × √3)R30° phase and the more complex (√7 × √7)R19.1° phase. The (√3 × √3)R30° forms at lower temperatures and corresponds to a simple sulfur overlayer. The (√7 × √7)R19.1° phase (for simplicity we shall call it the √7 phase) has been observed upon annealing sulfurized surfaces. The sulfur coverage in this case is estimated to be 3/7. A theoretical analysis [92] shows that this corresponds to a single PdS atomic overlayer as proposed by Liu et al. [89]. The S atoms lie at slightly off-bridge sites and are slightly below the Pd atom plane.

A theoretical study of thiol adsorption by Carro et al. [88] considered the formation of the thiol layer on a (√7 × √7)R19.1° PdS layer and concluded that upon thiol adsorption, some Pd adatoms are extracted from the PdS layer. The thiols attach to these “extracted adatoms”.

In order to shed light on characteristics of thiol adsorption, we first studied [48] sulfur adsorption on Pd(111) since CLBEs for S on the √7 phase were not known. Thereafter, dodecanethiol (C12T) adsorption was performed on pristine Pd. We also investigated C12T adsorption on both presulfurized and preselenized Pd surfaces, which allowed us to highlight characteristics of adsorption on a chalcogenide interface and distinguish between the thiol S and the interface chalcogen atom. We briefly summarize here the main findings of this study.

**Figure 3:**
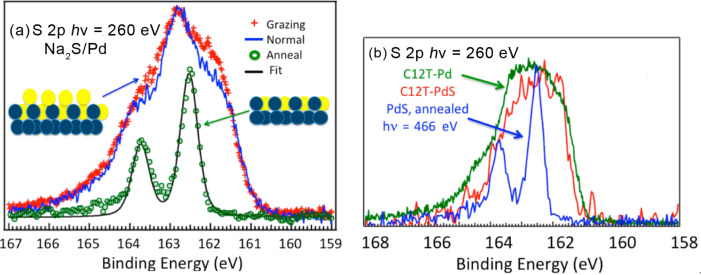
(a) XPS spectra [48] in the S 2p region before (blue, normal emission; red, grazing emission) and after annealing at 450 °C (green line). (b) Comparison of S 2p spectra [48] for the √7 PdS surface after annealing (blue line), PdS with adsorbed C12T molecules (red line), and clean Pd(111) with C12T molecules (green line). Figure adapted with permission from [48], copyright 2014 American Chemical Society.

Figure 3a shows the measured XPS spectrum in the S 2p region. As observed in previous investigations, the spectrum is broad, without well-defined features. It was fitted with two main doublet components at 161.71 and 162.45 eV and significantly smaller structures at 163.26 and 164.26 eV. These values were similar to previous reports [46–47,88] in a low resolution study on polycrystalline Pd with components at 162.1 eV (or 162.3 eV) and 162.9 eV (or 163.2 eV). It was suggested [88] that the thiolate CLBE has the higher binding energy, whereas the lower one corresponds to the sulfide phase; although in many cases of thiol adsorption on metal, the thiolate S 2p CLBE is close to 162 eV.

The S 2p spectrum for a sulfurized surface prepared in a Na_2_S aqueous solution followed by annealing to a few hundred Celsius (Figure 3b) was studied. The initial spectrum was found to be broad with a lower energy component. From angular measurements (i.e., normal and grazing emission), it was concluded that this component corresponds to atoms in an upper layer. Upon heating, essentially only one doublet component is left in the spectrum as shown in Figure 3b. LEED measurements indicate that under this condition, a well-defined √7 phase exists. A comparison with the thiol spectrum in Figure 3a then suggests that if this is really the underlying sulfide layer, the thiolate component lies at lower binding energies.

To explore this further, the √7 phase PdS surface was first prepared and then exposed to C12T. The result was remarkably similar to the one for direct C12T adsorption. Therefore, the fits to the C12T/PdS spectra suggest that the thiolate peak lies at 161.8 eV. This results in the PdS S 2p peak being split into two main components at 162.35 and 162.96 eV, suggesting a restructuring occurs in the PdS layer (as would be expected from the model of Carro et al.) [88]. An important aspect in XPS analysis is potential radiation damage to molecular films. This was carefully checked and was found not to affect the above conclusions (see original publication [48] for details).

**Figure 4:**
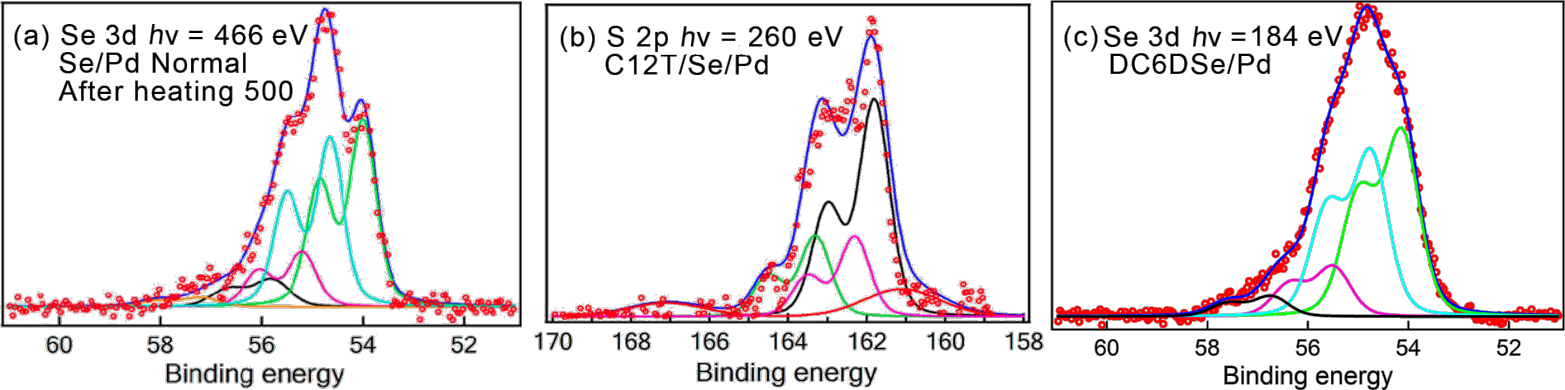
(a) XPS in the Se 3d region [48] after initial selenization of Pd with atomic selenium and heating to 500 °C. (b) XPS spectrum [48] in the S 2p region after C12T adsorption on the selenized surface (see text). (c) XPS in the Se 3d region after adsorption of DC6DSe (this work). Figure adapted with permission from [48], copyright 2014 American Chemical Society.

In a final twist in this investigation, we similarly prepared a selenized Pd surface in a Na_2_Se solution and later adsorbed the thiols onto this surface [48]. This was done in order to distinguish between the thiol S and the interface chalcogen atom. The Se 3d XPS spectra of the selenized surface, after annealing to 500 °C, are shown in Figure 4a. Interestingly, in this case, on both the initial selenized surface and after annealing, we obtained multicomponent spectra. In both cases, from angular emission measurements the lower energy feature appears to correspond to outer lying Se atoms. The LEED results for the annealed surface [48] were akin to the √7 PdS phase, except that multiple spots suggested a more complex structure with widely spaced, rotated domains.

C12T adsorption on the annealed, selenized surface resulted in changes in intensity of the Se 3d features: a clear decrease of the outer lying Se 3d peak was especially noticeable, indicating changes in the layer occurred. The S 2p XPS spectrum (Figure 4b) had a prominent doublet at 161.8 eV, as was deduced for the PdS surface case, supporting the attribution of the peak to thiolate for C12T adsorption on Pd and PdS. The higher energy component was attributed to X-ray damage effects, while the 162.3 eV component may be a different thiolate energy at a different adsorption site.

Measurements were also more recently performed for C6DSe adsorption on pristine Pd(111). The Se 3d XPS spectrum is shown in Figure 4c. As for the case of alkanethiol adsorption, the spectrum is broad. Its shape can be reproduced by fitting with two main components at 54.1 eV and 54.8 eV in addition to two smaller ones at 55.5 and 56.7 eV. The spectrum is remarkably similar to that corresponding to atomic Se adsorption. Although a detailed study for C12T was not performed, this similarity strongly suggests that we are dealing with C6DT dissociation and formation of a PdSe interface, possibly with molecular adsorption on this interface layer.

### Alkaneselenide and Se on Ni

The study of thiol and selenol SAM adsorption on Pd was extended here to the study of C6DSe adsorption on Ni and complemented by an investigation of Se interaction with Ni(111). We looked at Ni, since amongst other uses it can be employed as an electrode material. Additionally, Ni nanoparticles [93–97] are an example of magnetic nanoparticles [95] that are useful as catalysts [96], in magnetic fluids, as well as for binding and even magnetic separation of proteins [97]. As for other metals, undesirable oxidation has led to research into protection by chalcogenide SAMs, and while thiol adsorption has been investigated in some works [98], selenium head group molecule adsorption requires further study.

High-resolution XPS spectra were acquired on the Ni(111) surface selenized in the aqueous Na_2_Se solution and also on the annealed selenized sample. LEED measurements were performed to ascertain existence of ordered phases on the annealed surface. We will only focus on the main Se 3d results here, but results of the other XPS and LEED measurements are given in Supporting Information File 1, Figures S1–S3.

**Figure 5:**
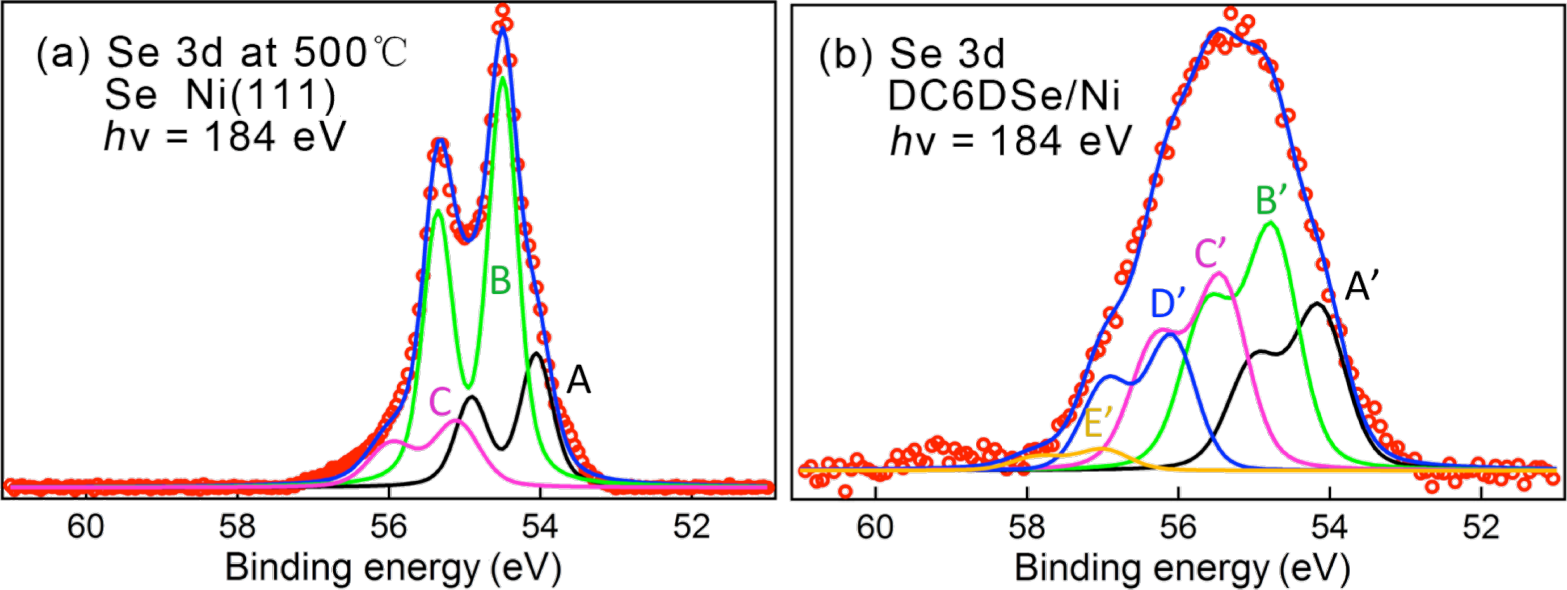
XPS in the Se 3d region after (a) initial selenization of Ni with atomic selenium and heating to 500 °C and (b) after adsorption of DC6DSe on Ni. See Supporting Information File 1, Tables S1 and S2 for peak positions.

The XPS spectrum in the Se 3d region after annealing the sample to 500 °C is shown in Figure 5 along with fits using Voigt contours (Supporting Information File 1, Table S1). The initial broad spectra obtained after immersion into solution and after heating to 300 °C are given in Supporting Information File 1, Figure S2 and Table S1. As shown in Supporting Information File 1, Figure S2, the spectrum peak positions shift and narrow upon heating. Heating to 500 °C leads to further decrease in width of the spectrum, which retains its main “B” component and has smaller features that appear to be remnants of contributions from differently coordinated Se atoms observed at lower temperatures (see Supporting Information File 1, Table S1). Indeed, LEED measurements on the heated samples show complex patterns that evolve with temperature (Supporting Information File 1, Figure S3).

High-resolution XPS measurements were performed for C6DSe adsorption from a millimolar solution in ethanol for an incubation time of one hour. Figure 5b shows the Se 3d region spectra for Ni(111). The spectrum, as was observed for Pd, is rather broad and can be fitted with several doublet components as shown in the figure (see Supporting Information File 1, Table S2). This suggests that here, as previously reported for thiol SAMs on Ni, we deal with Se–C bond breaking processes.

The attribution of the different components is challenging. In alkaneselenide adsorption on Au, the Se 3d_5/2_ CLBE of selenol for well-ordered SAMs is found to be close to 54.6 eV [32,37,39,43]. Here, however, as seen in Figure 5a, this energy corresponds to that of atomic Se on Ni for the case of an annealed surface (peak B). This comparison would lead to the tentative attribution of the two lower CLBE components (Figure 5b, A’ and B’) to the presence of atomic Se due to Se–C bond scission. The higher energy peak, C’, could then be due to molecular Se, and the peak D’ could possibly be due to a different atomic Se species.

As in the case of thiols on Pd, it is possible that in the initial stages of alkaneselenide adsorption we observed Se–C bond scission with the formation of a passivated Se–Ni surface. Thereafter, C6DSe adsorption occurs, which also leads to modifications in the structure of the Se–Ni surface layer as was the case for Pd. The attribution of the different components in the spectrum is only tentative and a more in-depth analysis is still necessary.

### Thiophene-family compounds

Thiophene derivative, π-conjugated systems have attracted much attention in molecular electronic applications [63–65,99–101] because of their interesting properties, structural versatility, intrinsic charge transport behavior with high carrier mobility, and high light harvesting efficiency. Their use includes application in field effect transistors, solar cells and light emitting diodes. A number of studies have been devoted to the assembly of these molecules on metallic electrodes [102–104] and in particular on gold surfaces [56–65]. They reveal peculiar features and differences in adsorption characteristics.

Evaporative assembly of thiophene onto Au(111) by Nambu et al. [56] at low temperatures (around 120 K) shows initial adsorption in a lying down configuration and then a transition to a more standing up configuration, until at high exposures a multilayer is formed. The S 2p_3/2_ CLBE for multilayer thiophene is about 164.5 eV whereas at monolayer coverage it was found to be 163.8 eV. This difference in position is due to the S Au surface interaction in the monolayer of the molecularly adsorbed thiophene. In liquid phase adsorption, on the other hand, a single doublet is observed with S 2p found at 162 eV, and this has been attributed to S–C bond scission leading to the appearance of a thiolate sulfur of an alkene chain. A shift to higher energy and broadening of the C 1s peak is also observed. In NEXAFS measurements, for molecular adsorption in vacuum, the spectrum is characterized by a sharp peak at about 285 eV related to the π*1 orbital of thiophene, which disappears for liquid phase adsorption, indicating breaking of the thiophene molecule.

The work of Noh and Hara’s groups [57–58], however, found a main peak with S 2p_3/2_ CLBE near 162 eV and a smaller extra feature at 161 eV in liquid phase adsorption. The latter was tentatively attributed to atomic sulfur due to complete desulfurization of thiophene or possibly to adsorption at an alternative adsorption site. For thiols, this question of alternative adsorption sites was recently discussed by Jia et al. [27]. Interestingly, in the case of bithiophene adsorption [58], no significant dissociation was observed with the XPS spectrum composed of a single doublet S 2p at 163.4 eV. This led to the conclusion [58] that the adsorption state of the thiophenes depends on the number of units in the thiophene oligomer. Yet another conclusion was reached by Liu et al. [60], who reported for thiophene a S 2p_3/2_ binding energy of 163.4 eV for low temperature adsorption, whereas at room temperatures it was close to 161 eV. Thus there exist rather different accounts on thiophene adsorption on Au.

**Figure 6:**
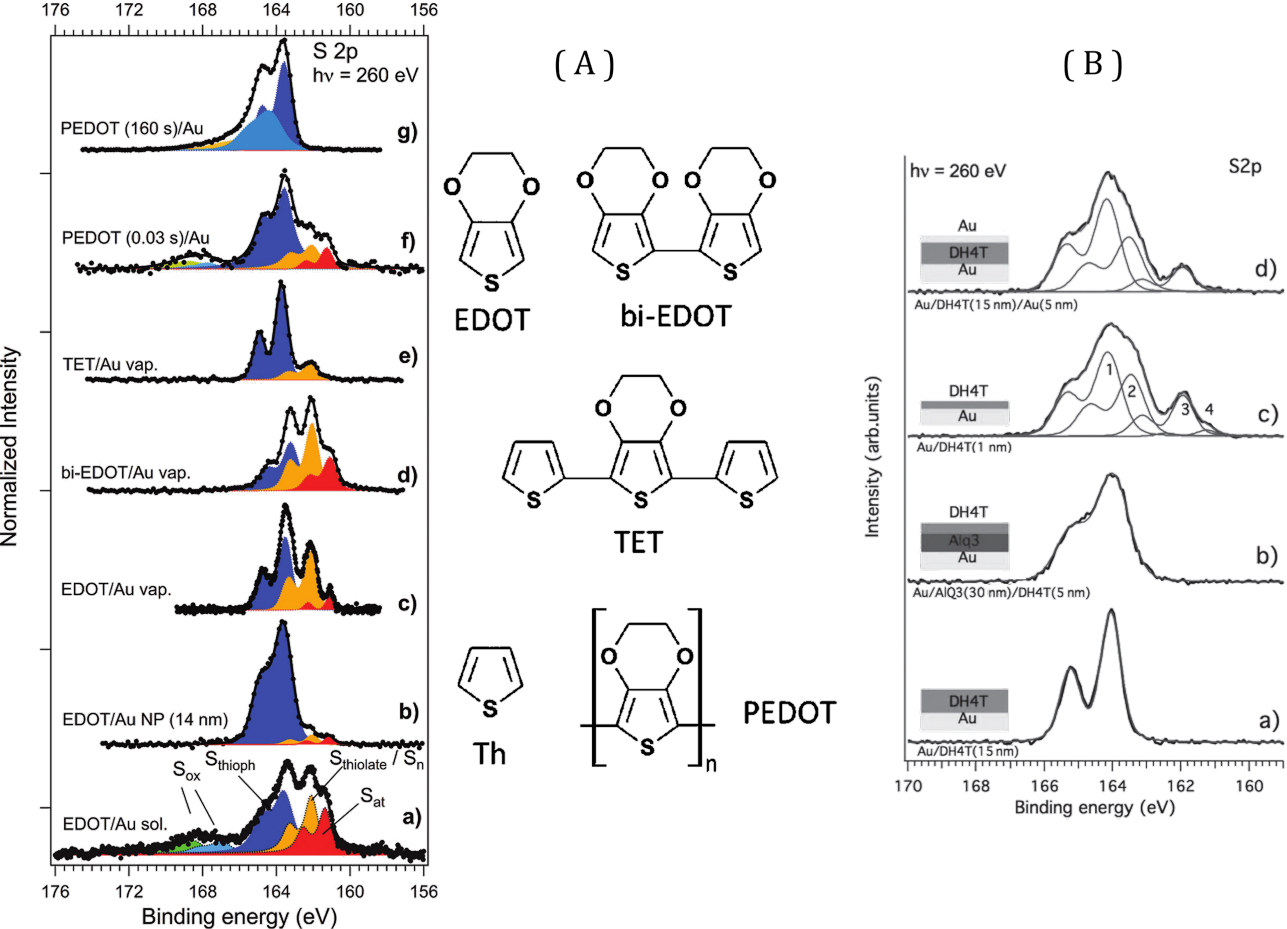
(A) EDOT-related molecules and XPS S 2p spectra for these cases [62]. Figure adapted with permission from [62], copyright 2011 American Chemical Society. (B) XPS spectra for DH4T [63] on gold for different cases of deposition as indicated for each curve. In this panel, the spectra refer to: a) 15 nm thick layer of DH4T on Au, b) DH4T layer on AlQ3 film on Au, c) a 1 nm DH4T layer on Au and c) a 5 nm layer of Au evaporated onto a 15 nm DH4T layer on Au. Figure adapted with permission from [63], copyright 2014 John Wiley & Sons, Inc.

Studies of 3,4-ethylenedioxythiophene (EDOT) and its derivatives [62] (Figure 6a) on polycrystalline Au, Au(111) and Au nanoparticle (AuNP) surfaces from vapor phase and solution has also revealed complex S 2p spectra (Figure 6a) with components corresponding to molecular adsorption and the appearance of thiolate and possibly atomic sulfur. Thus, these components were observed with various degrees of intensity for EDOT, bi-EDOT, 3’,4’-ethylenedioxy-2,2’:5’,2”-terthiophene (TET), and the polymer (PEDOT). The dissociation process would not, in this case, depend on the number of units.

A recent detailed study [63] was performed for the case of α,ω-dihexylquaterthiophene (DH4T) which, because of its high carrier mobility [63], is of great interest for organic electronics. We studied the assembly on Au surfaces from photoemission and XPS spectra in the S 2p region as shown in Figure 6B. For a thick, DH4T layer one observes the S 2p_3/2_ peak related to thiophene rings at about 164 eV (peak 1), while for the thin layer, the same feature shifts to 163.4 eV (peak 2). This is due to the presence of thiophenes at the close interface with Au. One also observes features at about 162 eV (peak 3) and 161 eV (peak 4), attributable to the strong reaction, leading to S–C bond breaking and the appearance of thiolate (peak 3) and possibly atomic S (or possibly molecules at a different adsorption site; peak 4). A similar spectrum is obtained when Au is evaporated onto the thick DH4T layer [63] and reactions likely occur at the diffuse interface. Indeed, the partial penetration of Au into the layer may occur, as this has been observed in metal evaporation onto organic samples. This was noted for Au, Ag and Cu electrode evaporation onto other thiophenic derivatives such as poly(3-hexylthiophene), where penetration of the metal into the layer was also suggested to occur [64–65].

**Figure 7:**
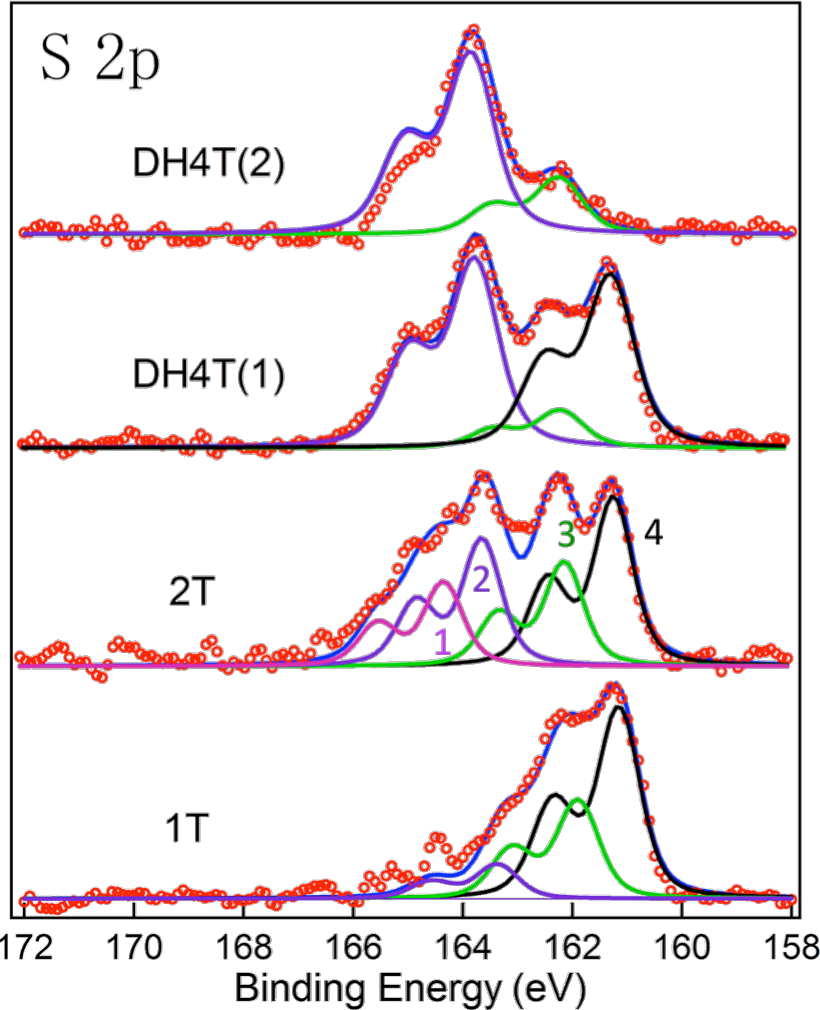
XPS S 2p spectra for 1T, 2T and DH4T adsorption on Au films on mica. The DT spectra are shown for two different samples prepared in the same manner.

New experiments were performed on adsorption of thiophene (1T), bithiophene (2T) and DH4T on Au(111) surfaces produced by evaporation of Au onto mica. Several sets of measurements were performed with variable results. The S 2p XPS spectra are shown in Figure 7 along with fits using Voigt contours. They show the existence of multiple doublet components with S 2p at 163.5, 162 and 161.5 eV, corresponding to: a) peak 2 - the thiophene molecule interacting with Au, b) peak 3 - a thiolate S of an alkene chain of the broken thiophene molecule and c) peak 4 - a large component either due to atomic sulfur or an alternative adsorption site of the molecules, respectively. Here we use the same notation as in Figure 6B [63]. For 1T we only see components corresponding to fragments from dissociation. In the case of 2T and DH4T, there is a component corresponding to adsorbed thiophene interacting with Au. The 2T result differs from that reported earlier [58], where the XPS study concluded that there was no dissociation. We were able to obtain such spectra quite systematically. Finally, for 2T the spectrum was fitted with a higher energy component (peak 1) ascribed to thiophene not interacting with Au, and for which the CLBE is similar to the one found for a thick molecular layer of thiophene (Figure 6B, peak 1).

The DH4T spectrum shows the same components as in the preceding study, albeit with different relative intensities and without the multilayer component (peak 1, Figure 6B). Here, for DH4T, we show data for two different samples, prepared under the same conditions, but which give very different results. We emphasize this variability, which we attribute to differences in the surface characteristics that can lead to differences in reactivity and changes in the relative intensity of components related to dissociation channels. This can also explain the difference between the characteristics of the spectra shown here and earlier works. Note also that as mentioned above and shown for thiols [27], adsorption of the S atom of the alkene chain at different adsorption sites could lead to different CLBEs close to 162 and 161.5 eV. This may depend on the order and packing density of the molecules.

From these results, one sees that on Au (which is considered to be nonreactive), thiophene and its various derivatives undergo S–C bond scission. The catalytic activity and electron transfer processes for Au have been extensively investigated in recent years and are shown to be quite large on nanoclusters [105–110]. This has been related to low coordination sites [109–110] and to the density of steps and different kinds of surface defects. Variability, to the extent of dissociation processes, could thus be expected depending upon the structure of the surface.

### Selenophene on Cu(111)

Selenophene (Seph)-based compounds are considered interesting alternatives [111] to thiophene. Selenophene adsorption on Au surfaces in vacuum and from liquid was reported by Kondoh et al. [112], who, as for thiophene, reported molecular adsorption of selenophene in UHV, but dissociative adsorption from liquid phase adsorption. This was deduced from changes in the Se 3p peak positions and strong differences in NEXAFS spectra. In adsorption in UHV, a strong peak due to the selenophene π orbital was observed, whereas it was very strongly reduced for liquid phase adsorption.

**Figure 8:**
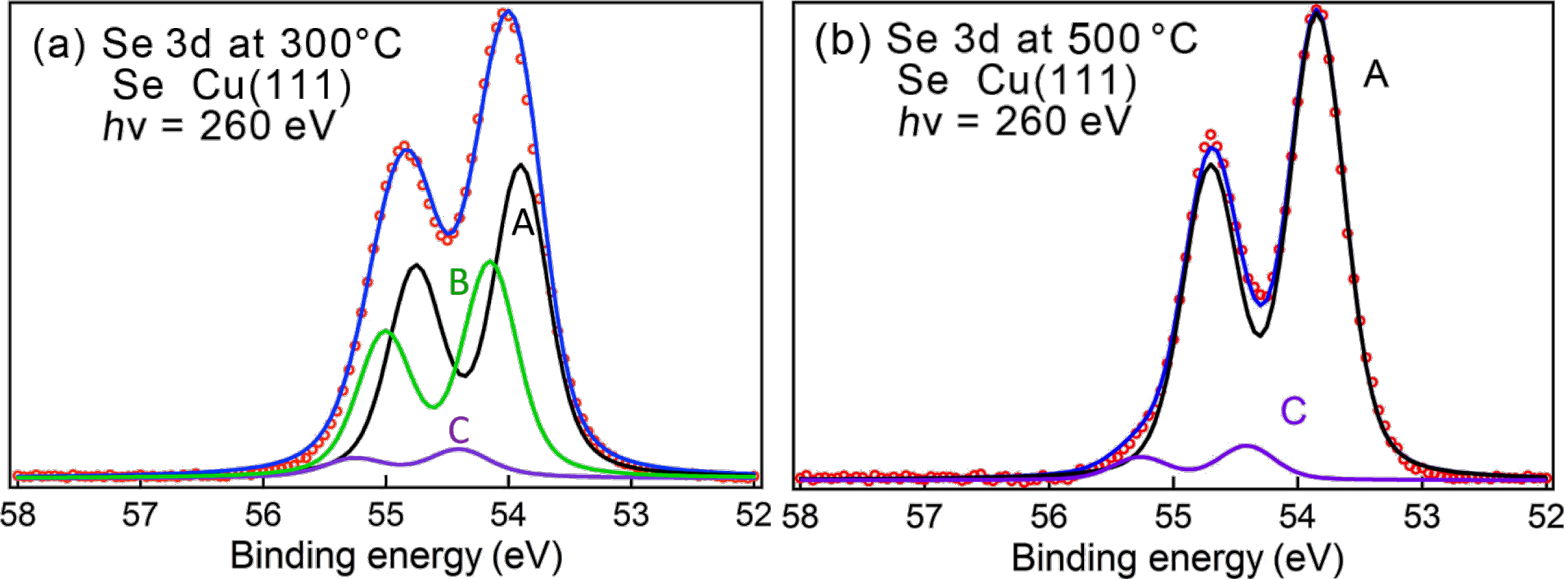
Se adsorption on Cu(111) from a Na_2_Se solution and after heating to the indicated temperatures.

Along the lines of the other studies, we first examined Se adsorption on Cu(111) from a Na_2_Se solution, with the objective of determining the Se CLBEs on Cu surfaces for ordered sub-monolayer structures. The photoemission data concerning Cu 2p and Cu 3p levels and the Cu Auger results are very similar to the case of sulfur adsorption [82] and we do not go into them here. The XPS spectrum in the Se 3d region after initial adsorption is broad with the Se 3d_5/2_ peak at 54.3 eV. The resulting spectra after annealing to 300 and 500 °C are shown in Figure 8, which includes fits using Voigt profiles. After the first annealing step, two main components are observed with Se 3d_5/2_ CLBEs of 53.9 eV (A) and 54.2 eV (B) and a small feature (C) at 54.5 eV, indicating differently coordinated Se atoms. After the second annealing, component A dominates the spectrum. These changes are also reflected in LEED images, where rather different structures are observed after annealing. These will not be discussed in this short report.

**Figure 9:**
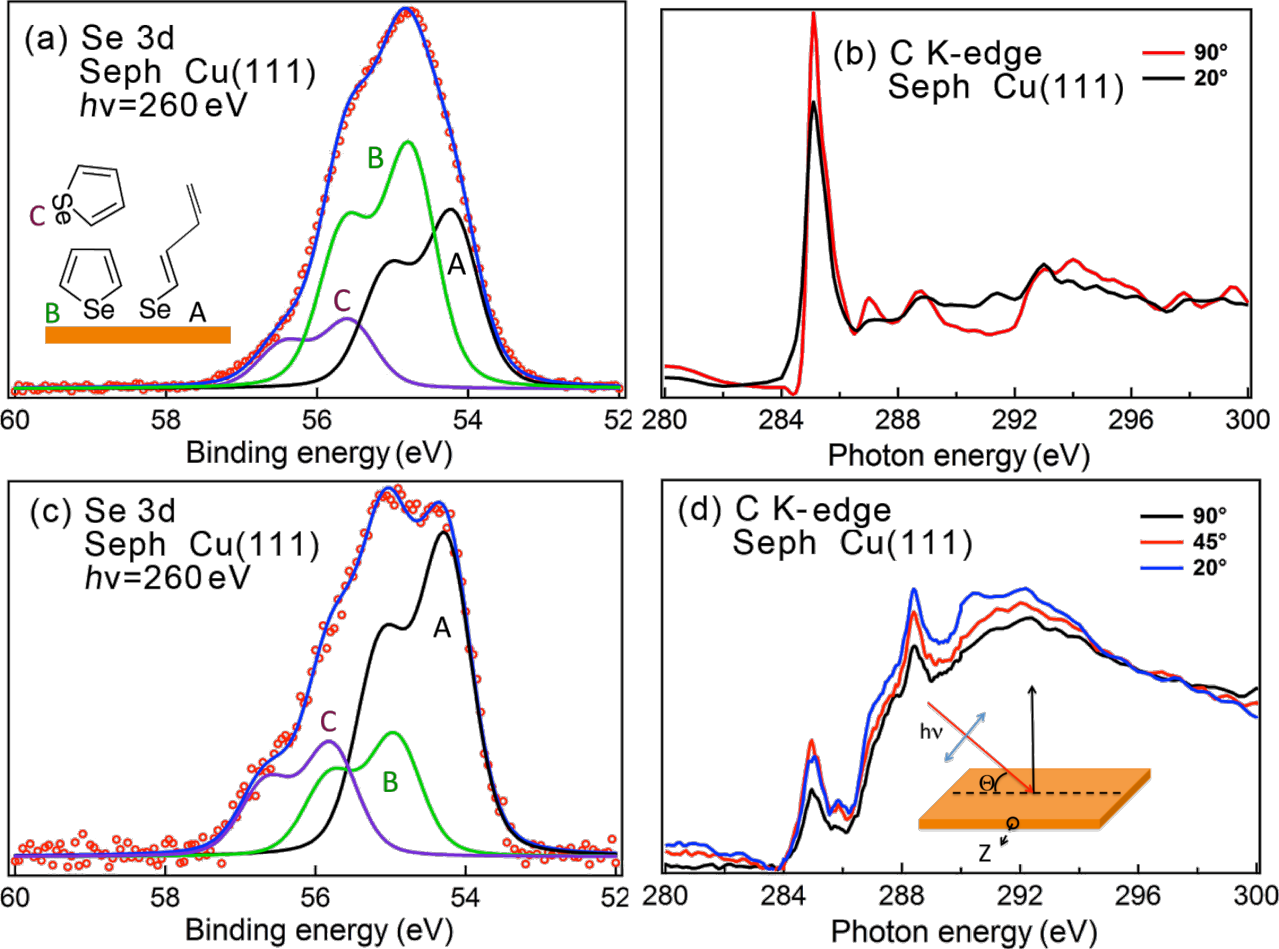
Selenophene (Seph) adsorption on Cu(111). (a,c) Se 3d spectra and (b,d) NEXAFS spectra for the indicated angles.

High-resolution XPS and NEXAFS spectra for selenophene adsorption onto Cu(111) are shown in Figure 9. We show the results of two sets of measurements (Figure 9a,b and Figure 9c,d, corresponding to what we later call Sample 1 and Sample 2, respectively). In both cases, the Cu(111) surface preparation and incubation in pure selenophene was performed in the same manner.

The XPS spectra display a considerable variability as for the thiophene case. In general, a broad Se 3d spectrum is observed, which can be fitted with three with Se 3d components located at 54.3 eV (A), 54.85 eV (B) and 55.8 eV (C). The relative intensities of these components vary greatly between the two measurements sets.

On the basis of a comparison with the Se 3d spectra for atomic Se adsorption on Cu and with the CLBE of selenol adsorption on Au, we could ascribe the different components in the Se 3d spectrum to: a) presence of Seph Se 3d components, b) Seph dissociation, leading to alkene-selenol-like CLBEs and c) possible atomic Se or molecules adsorbed on different adsorption sites. The CLBE for atomic Se adsorption lies at lower energies after high temperature annealing (Figure 9), but we cannot entirely rule out its presence in this spectrum, because one does see higher energy components at the lower temperature.

Further information comes from NEXAFS spectra shown in Figure 9b,d. In Figure 9b, for Sample 1, the NEXAFS spectrum is dominated by a peak at 285.5 eV ascribable to the π*1 orbital of selenophene. For Sample 2 this peak is much weaker. We also see that for Sample 1 in the XPS 3d spectrum, the B peak is most intense, whereas for Sample 2 it is the A peak that dominates the spectrum. In this case, there is also a more prominent peak at 288.4 eV and a shoulder at 292.2 eV, which could be related to the alkene chain. We rule out that this difference in Sample 2 is due to X-ray irradiation effects since measurements as a function of scanning did not reveal any significant changes (see Supporting Information File 1, Figure S4).

On the basis of these results, it would seem reasonable to ascribe peak B to selenophene interacting with the surface. Furthermore, peak A can be related to Se adsorption corresponding to one Se–C bond breaking, leading to an alkene chain appearance. Finally, the higher energy C component can be related to residual (after rinsing) selenophene remaining on the surface (e.g., on top of the rest of the molecular film) and not reacting strongly with Cu. This is schematically illustrated in the inset of Figure 9a. These attributions follow the scheme for thiophene described above. The peaks in the fit are somewhat broad, which may reflect presence of atomic Se at the lower energy end of the spectrum and also different bonding configurations of molecules. The NEXAFS spectra suggest, in both cases, that the molecules are either strongly tilted from the surface normal or that a large fraction of the layer is disordered.

Selenophene adsorption thus shows the possibility of dissociation with Se–C bond scission and underlines again the variability, which could be related with surface morphology, different probabilities of reactive bond breaking or different adsorption configurations.

## Conclusion

The results presented here show that in a number of cases in self-assembly of chalcogenide molecules on metal surfaces, dissociation processes are observed that correspond to chalcogen-atom/C-bond scission. While in case of the more reactive transition metals this may not appear surprising, these processes are also observed on the less reactive coinage metal surfaces, including gold.

In the case of the copper surface, earlier studies of alkanethiol and phenyl thiol adsorption did not reveal existence of any dissociation process, but we see that in the case of BDMT S–C bond scission is observed at room temperature. This occurs in the early stages of adsorption, corresponding to the lying down phase, leading to formation of a sulfurized surface on which molecules are later adsorbed after its passivation. There are, until now, no theoretical studies that would help to understand these differences and in what way the presence of the methylene unit promotes dissociation, as opposed to the case of alkane and phenyl thiols. Such studies in the early phase of adsorption would be most interesting.

In the case of Pd and Ni, we see that also chalcogenide molecule adsorption is accompanied by formation of a chalcogenized interface layer on which molecules are then adsorbed. One can expect that capping nanoparticles with these molecules would lead to formation of metal–metal chalcogenide, core/shell nanoparticles, which has been shown to have interesting specific properties.

In the case of chalcogenophene molecules, we also observed that even for gold, S–C (Se–C) bond scission occurs, leading to opening of the ring and loss of aromaticity. This interrupts the π-electron system and impairs charge transport along the chains, which is a problem in molecular electronics. There are indications of the appearance of atomic S/Se on the surface that corresponds to complete removal (dechalcogenation) of the molecules. It has been suggested that this may be accompanied by metallocycle formation [56,112].

In general, such dissociation processes leading to formation of chalcogenide interface layers accompanied by changes in molecular properties can adversely affect charge transport. In metal deposition on dithiol SAMs, which has been considerably discussed, the reaction with the metal with S–C bond scission would result in cutting the link with the rest of the molecule. In molecular electronics applications, this would result in a disruption of current flow.

In this work, we have strived to underline the variability in the adsorption results, where we see that under seemingly similar preparation conditions, quite different results are obtained with significant dissociation occurring in some cases, even though the preparation procedures appear to be reasonably good. We would relate this at least partly to surface morphology, since reactivity can be large at low coordination sites and depends on the density of steps and different kinds of surface defects. It is important to delineate this from the point of view of creation of metal contacts in organic electronic devices. This should be taken into account in general, including the case of evaporative deposition or contact printing [72] by transfer from a stamp.

We hope that this report will stimulate further investigations of these reactive processes and be useful to researchers dealing with these systems in various applications mentioned here.

## Supporting Information

File 1 Case studies of formation of chalcogenide self-assembled monolayers on surfaces and dissociative processes.
Selenide synthesis; XPS spectra for selenium on nickel and tables of Se 3d peak positions; LEED images for Se on Ni(111); X-ray damage verification for selenophene.
