# Supplementary material for: Case studies on the formation of chalcogenide self-assembled monolayers on surfaces and dissociative processes
Source: Beilstein J Nanotechnol. 2016 Feb 17;7:263–77. doi: 10.3762/bjnano.7.24 (PMC4778531; doi:10.3762/bjnano.7.24)
Supplement: File 1 — Case studies of formation of chalcogenide self-assembled monolayers on surfaces and dissociative processes. Selenide synthesis; XPS spectra for selenium on nickel and tables of Se 3d peak positions; LEED images for Se on Ni(111); X-ray damage verification for selenophene. [file Beilstein_J_Nanotechnol-07-263-s001.pdf]

Supporting information

for

Case studies on the formation of chalcogenide self-assembled monolayers on surfaces and dissociative processes

Yongfeng Tong[1,2,§], Tingming Jiang[1,2,3,§], Azzedine Bendounan[2], Makri Nimbegondi Kotresh Harish[4], Angelo Giglia[5], Stefan Kubsky[2], Fausto Sirotti[2], Luca Pasquali[3,5,6], Srinivasan Sampath[7] and Vladimir A. Esaulov[1*]

Address: [1]Institut des Sciences Moléculaires d'Orsay, UMR 8214 CNRS-Université Paris Sud, Université Paris-Saclay, F-91405 Orsay, France, [2]Synchrotron Soleil, L'Orme des Merisiers, Saint-Aubin, BP 48, F-91192 Gif-sur-Yvette Cedex, France, [3]Dipartimento di Ingegneria 'E. Ferrari', Università di Modena e Reggio Emilia, Via Vignolese 905, 41125 Modena, Italy, [4]Acharya Institute of Technology, Bangalore 560 107, India, [5]CNR-IOM, s.s.14, km 163.5 in Area Science Park, 34012 Trieste, Italy, [6]Physics Department, University of Johannesburg, P.O. Box 524, Auckland Park 2006, South Africa, and [7]Department of Inorganic and Physical Chemistry, Indian Institute of Science, CV Raman Avenue, Bangalore 560 012, India

Email: Vladimir A. Esaulov* - vladimir.esaulov@u-psud.fr

***** Corresponding author

[§] Equally contributing authors

Selenide synthesis; XPS spectra for selenium on nickel and tables of Se 3d peak positions; LEED images for Se on Ni(111); X-ray damage verification for selenophene.

Selenide synthesis

Dihexyl diselenide (DC6DSe) was synthesized by modifying an already reported procedure [1]. The reaction was performed under argon atmosphere. Hydrazine hydrate (0.3 mL) was added drop wise into a mixture of Selenium (22 mmol) and sodium hydroxide (33 mmol) in DMF (20 mL). After 15 min hexyl bromide (22 mmol) dissolved in DMF (20 mL) was added and the reaction mixture was stirred at room temperature for 8 h. After the completion of the reaction the mixture was treated with 100 mL water and allowed to stand for a few hours. The orange oily layer was separated and repeatedly treated with water to remove DMF. The product obtained was extracted in dichloromethane and was further purified by column chromatography (n-hexane, silica gel 60–120 mesh). The final yield is 69%.

Se adsorption on Ni(111)

XPS spectra in the Ni 2p, Ni 3p, and Se 3p regions as well as Ni Auger spectra and the valence band region are shown in Figure S1. The spectra after initial Se adsorption in solution are usually broad and display peaks due to O 1s and C 1s, which may be expected since the samples are exposed to water and then momentarily to air before introduction into the vacuum chamber. The Ni 2p spectrum is initially attenuated and the peak is shifted to higher binding energies: to 853.2 eV, a value close to that of nickel selenide [2], and shows features that can be associated with Ni^{+} and Ni^{2+} at 855.7 eV and about 861.3 eV After

heating, the spectra narrow and shift, similarly to the case of NiS [3]. The Ni $2p_{3/2}$ peak now lies close to the clean Ni position at 852.4 eV. A small change is also observed in the Auger spectrum, where the peak moves from 846.3 eV to 846.5 eV. The Ni 3p peaks shifts slightly to lower binding energies from 66.1 eV to 65.9 eV upon heating.

Concerning the valence band, just after deposition of Se, a decrease of Ni 3d structure below the Fermi energy is observed, along with the rise of Se related features [4]. After annealing, the emergence of pronounced emission just below the Fermi energy is indicative of a more metallic surface, as evidenced for NiS [5].

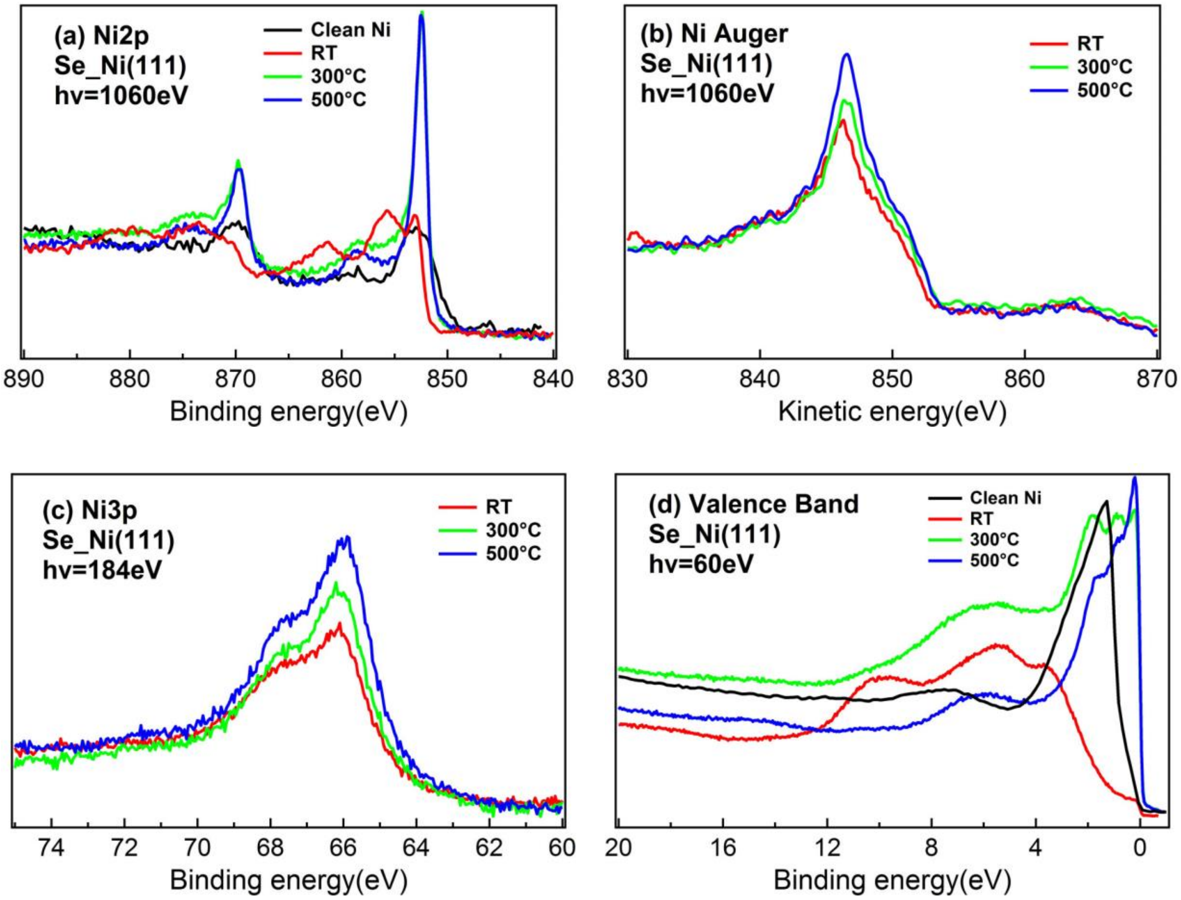

Figure S1: XPS spectra for Se adsorption on Ni(111) after immersion into the Na_2Se solution and annealing.

The Se 3d spectra are shown in Figure S2. After initial selenization the spectrum is broad and clearly composed of several components. A fit of the spectrum is shown using Voigt contours for the doublet with a spin orbit splitting of 0.86 eV and a relative intensity ratio of 0.66. The energies of the Se $3d_{5/2}$ component are indicated in Table ST1. The main component C has a value not far from the one reported for nickel selenide at 54.9 eV [2]. Upon heating the spectrum progressively narrows and is now dominated by the component B, along with some smaller intensity ones. Small shoulders at the low binding energy side lead us to introduce components A and D. Somewhat broader peaks at higher energies (E,F) are included to reproduce the high energy tail and are probably residues of the large features in the initial spectrum. With more heating, further narrowing occurs as shown in the Figure 5 of the main paper.

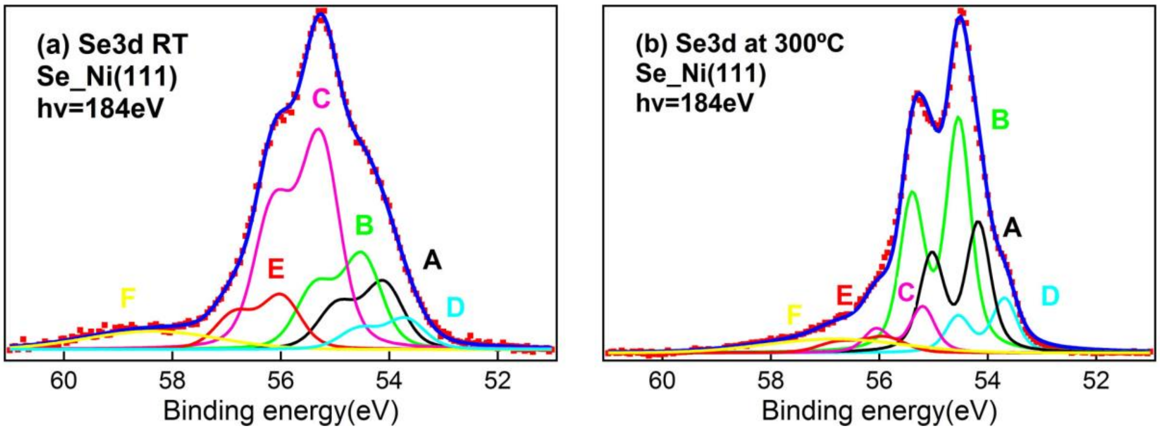

Figure S2: XPS in the Se 3d region after initial selenization of Ni with atomic selenium at room temperature (RT) and heating to 300 °C. Lines are fits as described in the text.

Table S1: Peak positions of Se $3d_{5/2}$ on Ni for adsorption from the Na_2Se solution (in eV).

Temperature	A	B	C	D	E	F
RT	54.1	54.3	55.3	53.7	56.0	58.0
300°	54.0	54.5	55.2	53.7	55.9	57.5
500°	54.0	54.5	55.2			

Table S2: Peak positions of Se $3d_{5/2}$ for DC5DSe adsorption on Ni(111) (in eV).

Temperature	A'	B'	C'	D'	E'
RT	54.1	54.7	55.5	56.0	57

The LEED images of the heated samples show complex patterns which evolve with temperature. Some images are shown in Figure S3 for reference. An STM study of these similar to the one performed for sulfur on Ni [6], would be interesting. We shall not go into any further details here, since this is not the focus of this report.

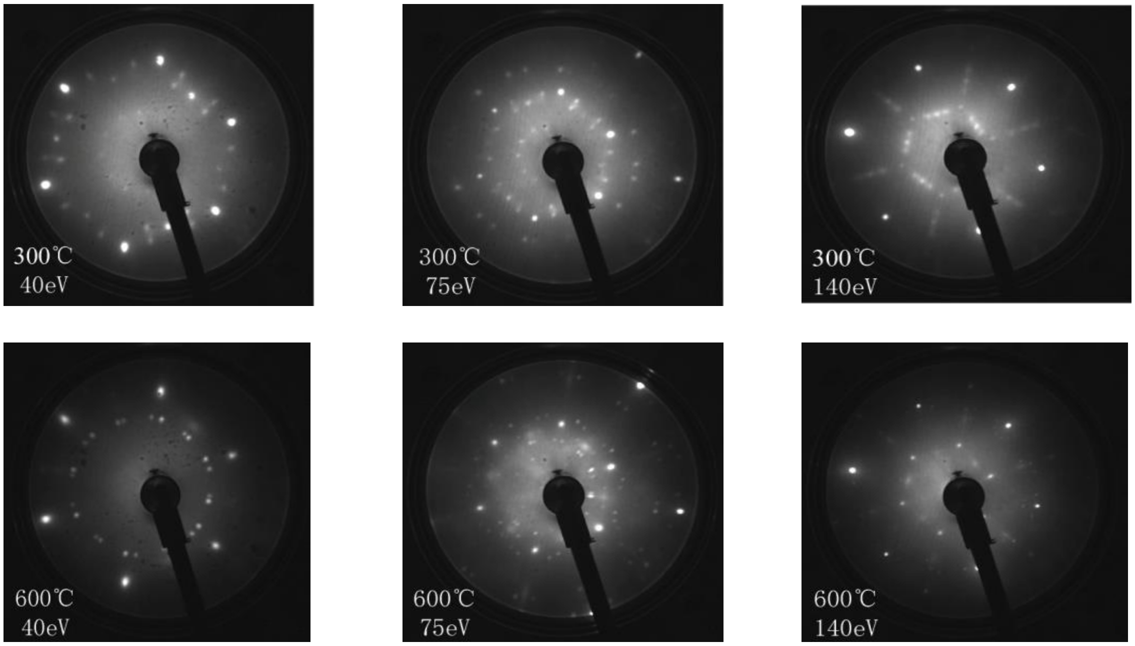

Figure S3: LEED images of the Ni–Se surface after annealing showing formation of complex ordered structures.

X-ray damage verification for selenophene on Cu

The effect of the X-ray beam on the selenophene layer on Cu is checked by acquiring spectra scan by scan. The color map of the intensities in the Se 3d spectrum and comparison of first and last scans do not show significant changes in shape.

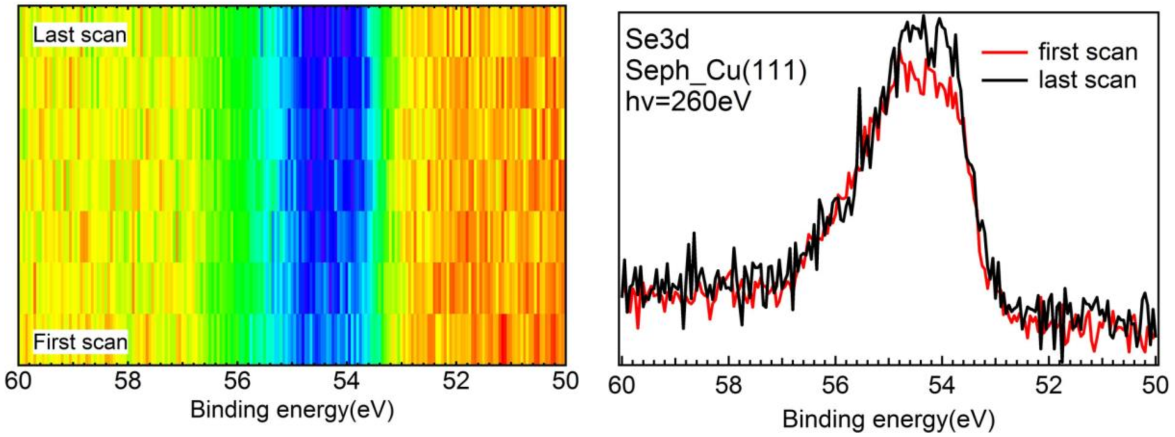

Figure S4: Color map of the intensities in the Se 3d spectrum for selenophene on Cu during scanning (left panel) and comparison of shape of first and last scans (right panel).

References

[1] Scianowski J., Tetrahedron Letters **2005**, 46, 3331-3334.

[2] Mandale A. B., Badrinarayan S., Date S. K. and Sinha A. P. B., Journal of Electron Spectroscopy and Related Phenomena, **1984**, 33, 61-72.

[3] Fontanesi C., Tassinari F., Parenti F., Cohen H., Mondal P.C., Kiran V., Giglia A., Pasquali L., and Naaman R., Langmuir, **2015**, 31, 3546-3552.

[4] Jacobi K., Muschwitz C.V., Solid State Communications, **1978**, 1978, 477-482.

[5] Krishnakumar S. R., Shanthi N., and Sarma D. D., Phys. Rev. **B 2002**, 66, 115105.

[6] Foss M., Feidenhans'1 R., Nielsen M., and Findeisen E., Johnson R. L. and Buslaps T., Stensgaard I. and Besenbacher F., Phys Rev **B 1994**, 50, 8950.
